# Abstract of the Proceedings of the Buffalo Medical Association

**Published:** 1870-03

**Authors:** B. H. Daggett

**Affiliations:** Secretary, Pro Tem.


ART. II.—*Abstract of the Proceedings of the Buffalo Medical Association.*

Tuesday evening, March 1st, 1870.

The meeting was called to order by Dr. Miner, President of the Association.

The Secretary, Dr. Johnson, being absent, Dr. Daggett was appointed Secretary.

After calling the roll, on motion of Dr. Samo, the reading of the minutes of the previous meeting was dispensed with.

Dr. Samo, the regular Essayist for the evening, was excused until the next meeting.

Dr. Miner, after apologizing for introducing to the attention of the profession a somewhat rare disease of the eye, and asking indulgence on account of its interest, not only to the Oculist but also to the general practitioner, presented an eye which he had recently enucleated. After enucleation, it had been placed in alcohol to harden its humors, and section now showed the nature of the disease for which it had been removed. He gave the following history of the case, and pointed out the connection between the symptoms and the pathological conditions observed. Mr. P., aged forty-eight, consulted him February 10th, 1870, by advice of Prof. Moore, giving the following account of his malady. "About three months since was attacked quite suddenly with pain in the eye, and very soon observed that I could not see in the outer direction; objects soon came to have a moon shaped appearance, or, one side was darkened by something intervening between the eye and the object seen; this increased so that in a few days I was blind in this eye. Pain has been very severe, and never relieved by any treatment yet given it. Have visited many physicians, some of whom have called my disease amaurosis, or said that I had disease of the optic nerve."

Upon examination, the globe was found to be soft; cornea atrophied, and semi-transparent; through the centre could be observed a white milky substance looking like pus in the posterior chamber; the anterior chamber was nearly obliterated, the iris in near proximity to the cornea. The whole globe was atrophied, and all its tunics together with its conjunctival covering deeply, injected. The other eye also sympathized considerably—vision was dim, and bright light painful, or rather very unpleasant. The disorganization of the eye being so complete and the pain so severe, he had no hesitation in advising enucleation of the globe, which was proposed and gladly accepted. The operation was made in the usual method and was followed by immediate and complete relief of all the severe symptoms. No attempt was made to determine the exact pathological condition previous to operation. The eye had been previously examined by an ophthalmic surgeon, but he mistrusted that no definite idea of the true nature of the disease had been gained.

At this time the opacity of the cornea and the loss of transparency in all the media wholly prevented any other than superficial examination. It was however sufficiently apparent what course to pursue, and the more complete examination and accurate diagnosis was deferred for observation, after due preparation of the globe, by the hardening process, to which it had been subjected.

Section of the globe, shows extravasation of blood between the choroid and retina separating these membranes throughout nearly one-half of their entire extent. This effusion undoubtedly produced at first great intra-ocular pressure, and might have caused the hardness of the globe, so characteristic of Glaucoma, that disease of the eye, which appears to depend upon hyper-secretion within the globe, and in its advanced stages is attended by a stoney hardness. In this case the pressure had displaced the lens, produced opacity of it, caused partial obliteration of the anterior chamber, and finally resulted in atrophy and softening of the globe; these changes having been the final result of extensive extravasation of blood within the globe.

In reply to an enquiry by Dr. Samo, of the probable cause of such effusion, Dr. Miner remarked, that extravasations of blood from accident, such as a blow upon the eye, or even from the concussion of a severe blow elsewhere are common. The choroid is the vascular and pigmentary tunic of the eye-ball, its structure is very delicate and its vessels easily ruptured, extravasation may, therefore, occur from diseases of the eye, which influence intra-ocular circulation, such as glaucoma, choroiditis, or any other disease which should influence the circulation in the delicate vessels of this tunic. Any sudden congestion—such as may arise from vomiting and coughing, might induce it, especially in diseased conditions; sudden relief of intra-ocular pressure, such as is given by making iridectomy in glaucoma, or by the operation of paracentesis for temporary relief of intra-ocular pressure is known to induce hemorrhage; also severe and protracted use of the eye is supposed to act as cause of extravasation of blood within the globe. In many cases, as in the one now presented, the cause is unknown. The blood may be effused, upon either side of the choroid, or into its tissue. If but slight it may produce but transient effect, the product being absorbed, while if of considerable quantity it must cause detachment of, or pressure upon the retina, in either case, producing loss of vision. Many times the effusion of blood into the choroid or between it and the retina may be clearly determined by the ophthalmoscope, but after change in the transparency of the refracting media is produced, no aid can thus be obtained. This case would have been an exceedingly interesting and instructive one, could it have been observed carefully from its first appearance until the time of operation, instead of which, the system was subject to various plans of medication, to suit the views of the practitioners who were in attendance, many of whom treated the case as amaurosis, that name under which was formerly grouped almost all the diseases of the eye resulting in blindness, so amusingly and aptly described by Von Græfe as that "shadowy and uncertain condition, in which the patient sees nothing, and the surgeon also, nothing." Effusions into the globe of the eye are usually of so limited extent, that they are slowly removed by absorption, rarely requiring enucleation or other operative interference; but the pain in this case was so severe, had continued so long, and seemed to be increasing rather than growing less, and the other eye now beginning to suffer from sympathy, he regarded enucleation as the most certain and safe method of procedure.

To the inquiry, if simple puncture, or section of the cornea would not have offered relief? He replied: that possibly it might have offered satisfactory relief; but that, even if in possession of the knowledge obtained by examination after removal, he would regard enuclation much more safe and certain; that by the present methods it was not more difficult or dangerous, and left as good, if not better foundation for artificial eye. But he did not know before operation, the nature or cause of the change which had taken place, and had reason to fear that nothing short of entire extirpation, if indeed that, would afford permanent relief. Irritations in the ciliary region, as in this case, are so liable to induce sympathetic inflamation in the healthy eye, that there should be no hesitancy, as there are no valid objections, to enucleation of the entire globe.

Diphtheria, Scarlatina, Typhoid Fever, Whooping Cough and Diarrhœa were reported as prevailing.

B. H. DAGGETT, *Secretary, Pro Tem.*

